# Comprehensive Chlorine Suppression: Advances in Materials and System Technologies for Direct Seawater Electrolysis

**DOI:** 10.1007/s40820-025-01653-z

**Published:** 2025-01-22

**Authors:** Cenkai Zhao, Zheyuan Ding, Kunye Zhang, Ziting Du, Haiqiu Fang, Ling Chen, Hao Jiang, Min Wang, Mingbo Wu

**Affiliations:** 1 State Key Laboratory of Heavy Oil Processing, College of New Energy, China University of Petroleum (East China), Qingdao, 266580 People’s Republic of China; 2 Beijing National Laboratory for Molecular Sciences, New Cornerstone Science Laboratory, College of Chemistry and Molecular Engineering, Peking University, Beijing, 100871 People’s Republic of China; 3 Key Laboratory for Ultrafine Materials of Ministry of Education, School of Materials Science and Engineering, East China University of Science and Technology, Shanghai, 200237 People’s Republic of China

**Keywords:** Direct seawater electrolysis, Oxygen evolution reaction, Hydrogen evolution reaction, Chlorine suppression, Seawater electrolysis system

## Abstract

Seawater electrolysis offers a promising pathway to generate green hydrogen, which is crucial for the net-zero emission targets. Indirect seawater electrolysis is severely limited by high energy demands and system complexity, while the direct seawater electrolysis bypasses pre-treatment, offering a simpler and more cost-effective solution. However, the chlorine evolution reaction and impurities in the seawater lead to severe corrosion and hinder electrolysis’s efficiency. Herein, we review recent advances in the rational design of chlorine-suppressive catalysts and integrated electrolysis systems architectures for chloride-induced corrosion, with simultaneous enhancement of Faradaic efficiency and reduction of electrolysis’s cost. Furthermore, promising directions are proposed for durable and efficient seawater electrolysis systems. This review provides perspectives for seawater electrolysis toward sustainable energy conversion and environmental protection.

## Highlights

- Rational design of chlorine-suppressing catalysts based on mechanistic insights.
- Overview of recent advances in cutting-edge seawater electrolysis systems.
- Discussion of challenges and potential directions for direct seawater electrolysis enhancement.

## Graphical abstract

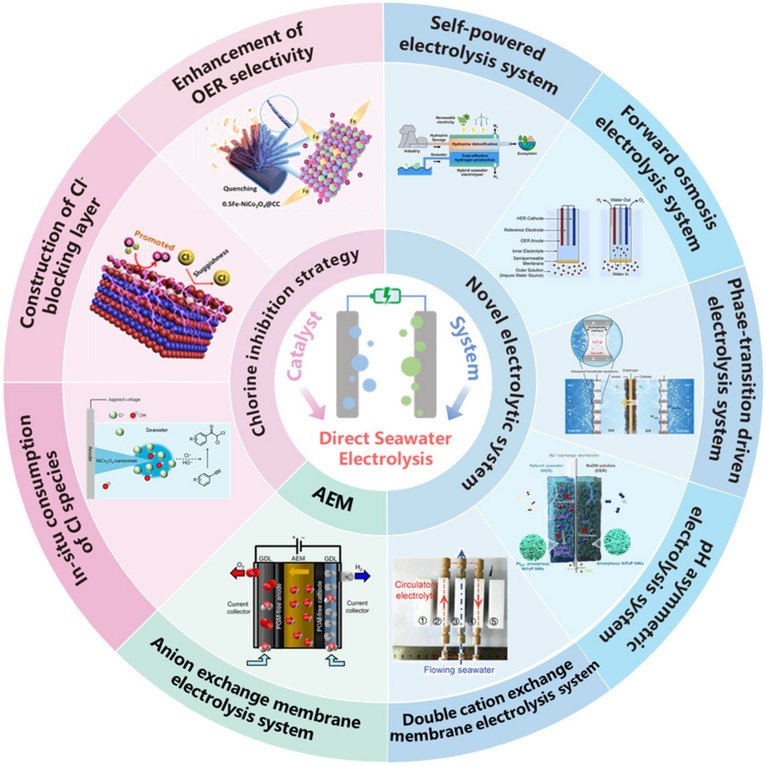

## Introduction

The renewable energy is experiencing rapid growth due to the global energy shortages and the environmental impact caused by fossil fuels [1–3]. The energy transitions are critical for alleviating energy crises, mitigating greenhouse gas emissions, safeguarding ecosystems, and promoting sustainable development [4]. Hydrogen is served as a clean and abundant energy carrier for energy storage and carbon dioxide emissions reduction [5, 6]. The demand of hydrogen is vigorously increasing due to energy demand and chemical reagent. The green hydrogen was generally defined as water electrolysis-derived hydrogen powered by renewable forces like wind and solar [7, 8], and it could generate minimal greenhouse gases during its production [9–11]. Therefore, the investment in renewable-powered water electrolysis technologies for green hydrogen production holds profound significance, as it drives the energy transition and supports carbon neutrality goals [11, 12]. However, water electrolysis, a key technology for clean energy generation, requires substantial freshwater resources. This raises concerns over the global distribution of freshwater and the exacerbation of water scarcity [13, 14]. In this context, the utilization of seawater for electrolysis presents a significant advantage by reducing the need for freshwater resources and leveraging the abundance of seawater. The seawater accounts for 96.5% of the Earth water and represented an almost inexhaustible resource and serves as a natural electrolyte, offering an ideal medium for the electrolysis process [15, 16].

There were two existing approaches for seawater electrolysis including indirect and direct methods [17]. Indirect seawater electrolysis requires seawater desalination before hydrogen production [18]. This approach mitigates the interference of seawater complex components during electrolysis. After extensive research, indirect seawater electrolysis has become a well-established and widely adopted technology [19]. However, the inherent need for an additional desalination step complicates the system, and the process does not fully eliminate residual ions. These residual ions can lead to corrosion or scaling, which deteriorates the electrolyzer performance by reducing efficiency and increasing maintenance requirements [20]. Moreover, desalination is energy-intensive, and it could bring high costs for the construction and maintenance of the complex systems in large-scale application [21–24]. In contrast, direct seawater electrolysis skips out the desalination stage, simplifying the hydrogen production process with reduced energy consumption and lower equipment and operational costs [25–27]. Furthermore, the vast availability of seawater resources globally makes direct seawater electrolysis a more efficient solution, particularly beneficial for arid coastal regions [19].

However, the complex composition of seawater presents significant challenges for direct electrolysis [21]. During this process, sharp pH fluctuations could occur at the electrode surface with increasing local pH levels near the cathode, directly causing cations such as Ca^2+^ and Mg^2+^ to readily precipitate [28]. These precipitates accumulate on the electrode and membrane surfaces, deactivating catalyst active sites and impairing catalytic efficiency [29]. Additionally, this fouling obstructs ion transport, reducing electrolyzer performance [30]. The presence of Ca^2+^ and Mg^2+^ also accelerates the corrosion, compromising the durability of the electrolyzer and associated components [31]. While direct seawater electrolysis circumvents desalination, pre-treating seawater by adding alkaline precipitants can mitigate precipitation and corrosion caused by these Ca^2+^, Mg^2+^ cations [32]. Furthermore, microfiltration of pretreated seawater effectively removes solid impurities and microorganisms, reducing the risks of flow channel physical blockages and catalyst poisoning [14]. This simplified treatment process enhances electrolysis efficiency and prolongs the operational lifespan of the system.

Another major challenge in direct seawater electrolysis is the competition between the oxygen evolution reaction (OER) and the chlorine evolution reaction ClER [14, 33–36]. Similar to freshwater electrolysis, seawater electrolysis involves two half-reactions: the cathodic hydrogen evolution reaction (HER) and the anodic OER, and the anodic OER has a standard thermodynamic potential of 1.23 V relative to the reversible hydrogen electrode (RHE) [37]. In practical applications, the relatively sluggish kinetics of the OER is the rate-determining step (RDS), thus constraining the overall efficiency of water electrolysis. The relevant electrode reactions are noted as following Eqs. (1–2) [38]:

1 $${\text{Acidic}}:\;2\hbox{H}_{2} \hbox{O} \to \hbox{O}_{2} + 4\hbox{H}^{ + } + 4\hbox{e}^{ - } \, \hbox{E}^{\theta } = 1.23 \, \hbox{V}$$

2 $${\text{Alkaline}}:\;4\hbox{OH}^{ - } \to \hbox{O}_{2} + 2\hbox{H}_{2} \hbox{O} + 4\hbox{e}^{ - } \, \hbox{E}^{\theta } = 0.40\,\hbox{V}$$

**Fig. 1:**
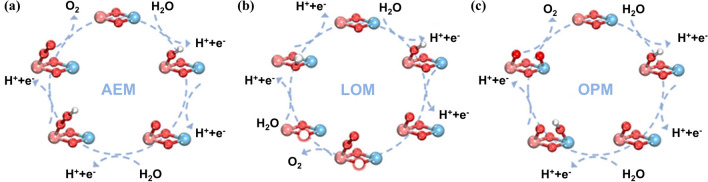
Three types of reaction mechanisms of OER. **a** Process of AEM involving the adsorption of OH^−^ without the participation of lattice oxygen in the reaction. **b** Process of LOM involving the participation of lattice oxygen in the reaction. **c** Process of OPM involving the direct coupling of oxygen radicals, leading to the formation of O_2_ without the generation of oxygen vacancies or the need for additional intermediates [45]

The traditional OER typically follows two primary mechanisms: the adsorption-enhanced mechanism (AEM) and the lattice oxygen mechanism (LOM) [39–41]. In the AEM, the reaction proceeds through the adsorption of OH^−^, formation of OOH^−^, and the eventual release of O_2_ (Fig. 1a). This process includes electron transfer between metal orbitals and an oxygen intermediate (O*), leading to a reduction in the oxidation state of the metal. This highlights the crucial role of the metal center in facilitating electron transfer.

In contrast, after the adsorption of OH^−^, the LOM mechanism promotes O–O bond formation via coupling lattice oxygen atoms. This process circumvents electron transfer to the external circuit (Fig. 1b). The LOM is distinguished by the hybridization of oxygen non-bonding states, which plays a crucial role in the transformation of peroxide (O_2_^2−^) into oxide (O^2−^), highlighting the redox activity of oxygen atoms during OER [42].

In the traditional OER, the AEM is hindered by limited catalytic activity due to scaling relationships, and the LOM faces instability from its fragile crystal structure. In contrast, the oxide pathway mechanism (OPM), as an emerging mechanism in the OER, facilitates direct O–O radical coupling without generating oxygen defects or additional intermediates, such as OOH [43, 44]. In this pathway, only O and OH act as intermediates, leading to catalysts that typically exhibit enhanced activity and stability, as shown in Fig. 1c [45].

In the alkaline media, the reaction pathways for the AEM, LOM, and OPM in OER are listed as following Eqs. (3–14) [46]:

AEM:

3 $$^{*} + \hbox{OH}^{ - } \to ^{*} \hbox{OH} + \hbox{e}^{ - }$$

4 $$^{*} \hbox{OH} + \hbox{OH}^{ - } \to ^{*} \hbox{O} + \hbox{H}_{2} \hbox{O} \, \left(\hbox{ l} \right) + \hbox{e}^{ - }$$

5 $$^{*} \hbox{O} + \hbox{OH}^{ - } \to ^{*} \hbox{OOH} + \hbox{e}^{ - }$$

6 $$^{*} \hbox{OOH} + \hbox{OH}^{ - } \to ^{*} + \hbox{O}_{2} \left(\hbox{g} \right) + \hbox{H}_{2} \hbox{O} \, \left(\hbox{ l} \right) + \hbox{e}^{ - }$$

LOM:

7 $$^{*} \hbox{O}_{\rm l} \hbox{H} + \hbox{OH}^{ - } \to ^{*} \hbox{O}_{\rm l} + \hbox{H}_{2} \hbox{O} \, \left(\hbox{ l} \right) + \hbox{e}^{ - }$$

8 $$^{*} \hbox{O}_{\rm l} + \hbox{OH}^{ - } \to ^{*} \hbox{O}_{\rm l} \hbox{OH} + \hbox{e}^{ - }$$

9 $$^{*} \hbox{O}_{\rm l} \hbox{OH} + \hbox{OH}^{ - } \to ^{*} \hbox{O}_{\rm l} \hbox{O} + \hbox{H}_{2} \hbox{O} + \hbox{e}^{ - }$$

10 $$^{*} \hbox{O}_{\rm l} \hbox{O} \to ^{*} + \hbox{O}_{2}$$

11 $$^{*} + \hbox{OH}^{ - } \to ^{*} \hbox{O}_{\rm l} \hbox{H} + \hbox{e}^{ - }$$

OPM:

12 $$^{*} + \hbox{OH}^{ - } \to ^{*} \hbox{O} + {\text{H}}_2{\text{O}} + \hbox{e}^{ - }$$

13 $$^{*} \hbox{O} + \hbox{OH}^{ - } \to ^{*} \hbox{OO}{ + }\hbox{e}^{ - }$$

14 $$2 \, ^{*} \hbox{OO} \to { 2 }^{*} + {\text{O}}_2 (\text{g}) + 2 \hbox{e}^{ - }$$

where ^*^ represents the metal site, and O_l_ denotes lattice oxygen atom.

In summary, the OER plays a critical role in water electrolysis. The OER involves a series of complex multi-electron and multi-proton transfer steps, necessitating multiple sequential chemical reactions on the surface of the catalyst. This inherent complexity involving multi-electron and multi-step reactions leads to sluggish OER kinetics, thus requiring significant energy input to drive the reaction. In contrast, the HER involves a simpler electron transfer mechanism, typically needing only a single electron transfer step to produce hydrogen, with substantially lower energy requirements [47–49]. Consequently, accelerating OER kinetics remains a central challenge in improving the overall performance of water electrolysis technology.

In direct seawater electrolysis, the abundance of Cl^−^ and scarcity of OH^−^ makes the ClER more favorable *versus* OER, enabling the ClER as a major competing side reaction for OER [50, 51]. Similar to OER and HER, the reaction pathways and products of ClER are influenced by factors such as the pH of seawater, reaction temperature, and the applied potential. The relevant reactions (Eqs. 15–17) are as follows [52]:

15 $$2\hbox{Cl}^{ - } \to \hbox{Cl}_{2} + 2\hbox{e}^{ - } \, \hbox{E}^{\theta } = 1.36 \, \hbox{V}$$

16 $${\text{Acidic}}:\;\hbox{Cl}^{ - } + \hbox{H}_{2} \hbox{O} \to \hbox{HClO} + \hbox{H}^{ + } + 2\hbox{e}^{ - }$$

17 $${\text{Alkaline}}:\;\hbox{Cl}^{ - } + 2\hbox{OH}^{ - } \to \hbox{ClO}^{ - } + \hbox{H}_{2} \hbox{O} + 2\hbox{e}^{ - }$$

**Fig. 2:**
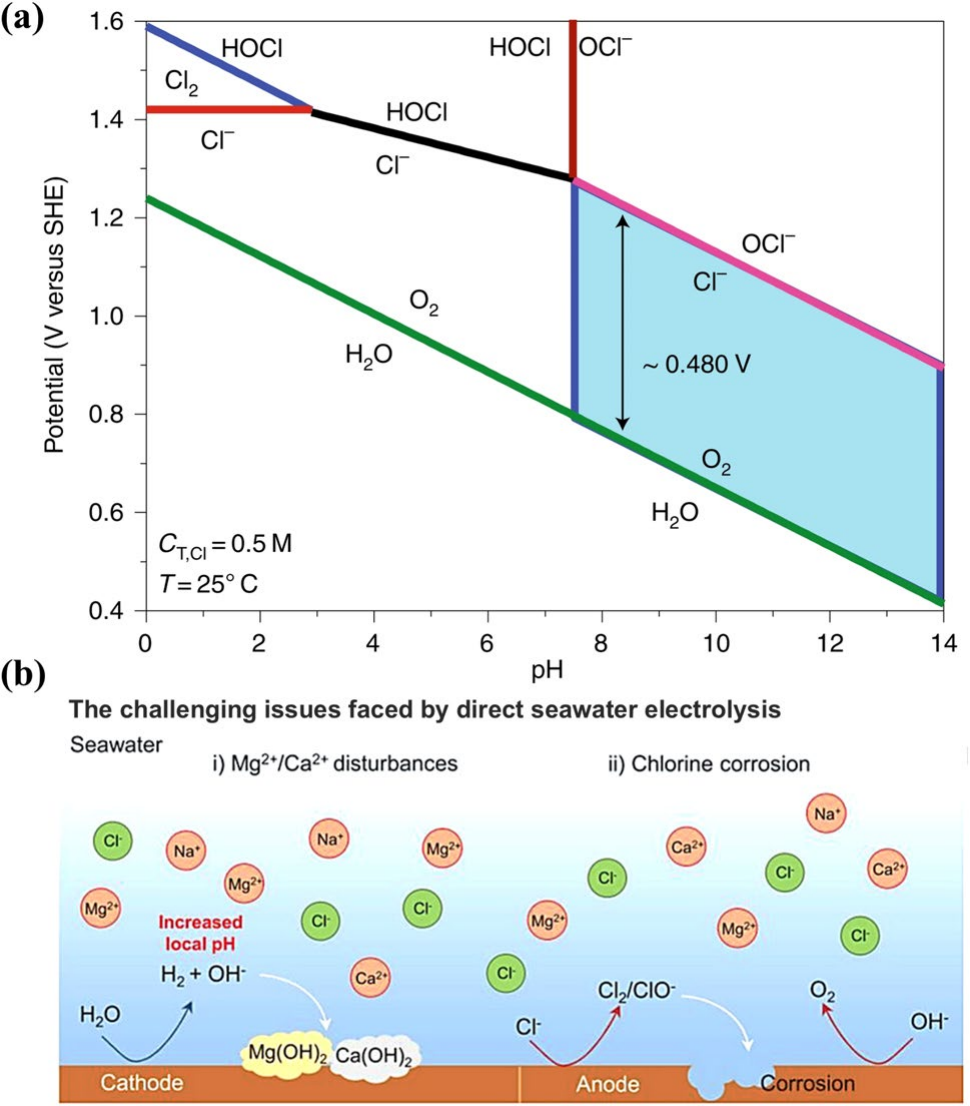
**a** Plot of electrode potential versus pH in 0.5 M NaCl aqueous solution [14]. **b** Fundamental issues faced by direct seawater electrolysis in hydrogen production [69]

In direct seawater electrolysis, the ClER can yield either chlorine gas or hypochlorite. Hypochlorite production is usually pH-dependent process, while formation of chlorine gas (Cl_2_) is independent of pH [53]. As depicted in the Pourbaix plot (Fig. 2a), the OER is more thermodynamically favorable than the ClER across a broad pH range [54]. Under the conditions of high pH, the potential difference between OER and ClER can reach up to 480 mV [14]. However, the sluggish kinetics of the four-electron transfer process in the OER increases its overpotential, diminishing its thermodynamic advantage in practical electrolysis [55–58]. Particularly, the narrow kinetic gap between OER and ClER with 0.13 eV makes ClER a significant competitive reaction in acidic environments. To elucidate the rate-determining step (RDS) of the OER, kinetic isotope effect (KIE) studies can be employed. By comparing the reaction rates of H_2_O and D_2_O in the OER process, the step involving the transfer of a proton (or deuteron) can be identified as the RDS [59, 60]. Specifically, a significant KIE would indicate that proton transfer is the RDS, providing insights into the role of the metal center in facilitating electron and proton transfer. Additionally, electrochemical techniques such as cyclic voltammetry (CV) and linear sweep voltammetry (LSV) can be used to probe the electron transfer kinetics at the catalyst surface, offering direct evidence of the RDS and the intrinsic activity of the catalyst [61]. Additionally, the lack of buffering ions in OER results in a localized drop of pH near the anode surface with aggravated overpotential and low efficiency [62, 63]. At high current density, the frequency and intensity of ClER may surpass OER, further undermining the desired OER dominance in the electrolysis [64].

Furthermore, the products of ClER including Cl_2_ and hypochlorite ions (ClO^−^) are highly oxidizing and corrosive to catalysts and metal components within the electrolyzer, as shown in Fig. 2b. These substances rapidly cause the degradation of catalytic activity, leading to a significant decline in the performance [65]. The corrosive nature of Cl_2_ and ClO^−^ also accelerates the degradation of metal components, causing both physical and chemical damage of flow plates [66, 67], which compromises the mechanical stability and chemical durability of the electrolyzer [68]. The degradation from ClER not only reduces the energy conversion efficiency but also increases the likelihood of unexpected electrolyzer failure, leading to higher maintenance costs and operational downtime. In the long term, the ClER poses a significant threat to the commercial viability and environmental safety of direct seawater electrolysis. Thus, it is essential for improving the stability and economic feasibility of this technology to develop effective strategies to suppress ClER and protect electrolyzers from Cl_2_ and ClO^−^.

In the context of globalization, the escalating energy demand and worsening environmental challenges have underscored the critical need for clean and sustainable energy solutions. Direct seawater electrolysis harnesses abundant marine resources to produce green hydrogen and is emerging as a pivotal pathway for energy transition. However, practical implementation of this technology encounters significant challenges, particularly in mitigating the detrimental effects of ClER on electrolysis efficiency and equipment integrity [70–73].

This review provides an in-depth analysis of recent advancements in electrode materials and seawater electrolysis systems, focusing on strategies to suppress the ClER and outlining future directions. The article first summarizes innovations in chlorine-suppressing electrode materials. Advances in material design, including electronic structure regulation, interfacial engineering, and local OH^−^ enrichment, have greatly enhanced the OER selectivity of electrodes. These strategies enable the OER to occur at lower overpotential and effectively minimize ClER. Furthermore, the review paper explores the concept and architectures of novel electrolysis systems. Membrane improvement technologies and the configurations of the reactor have successfully addressed issues related to Cl^−^-induced corrosion and undesirable side reactions in the seawater. Specifically, the optimized design of anion exchange membrane (AEM) electrolyzers enhances OH^−^ transport selectivity, thereby mitigating Cl^−^ corrosion and improving system durability. Moreover, emerging systems such as self-powered seawater electrolysis, forward osmosis-driven electrolysis, and phase-transition-driven electrolysis exhibit potential to lower energy consumption and boost Faradaic efficiency (FE) by incorporating renewable energy sources and novel mass transfer mechanisms.

This comprehensive discussion provides deep insights for current direct seawater electrolysis technology and future perspectives. As advancements in materials science, electrochemical technology, and system design continue, the commercial viability of direct seawater electrolysis is expected to improve, leading to a transformative shift toward a sustainable global energy framework.

## Chlorine Suppression Strategies

The high concentration of Cl^−^ in the seawater tends to form deposits on the electrode surface, which diminishes the density of active sites and subsequently impairs the overall efficiency of the electrolysis process [74, 75]. Additionally, the occurrence of ClER produces highly reactive chlorine gas (Cl_2_), which interacts with electrode materials, causing oxidation and corrosion. The Cl^−^ undergoes electron transfer reactions at the electrode surface, resulting in the generation of Cl_2_. During this process, the Cl^−^ loses electrons and becomes oxidized, which leads to the consumption of material from the electrode surface, causing both physical and chemical damage to the electrode [71]. In marine environments, the high concentration of Cl^−^ can accumulate on the electrode surface, potentially creating localized corrosive conditions that accelerate electrode degradation [71, 76]. This chlorine evolution not only depletes the electrode materials but may also result in the formation of a passivation layer, further compromising the performance of the electrode [77, 78]. Thus, it is imperative to design catalysts that are both highly efficient and resistant to seawater corrosion for improving electrolysis performance and advancing the commercial viability of seawater-based hydrogen production [79].

**Fig. 3:**
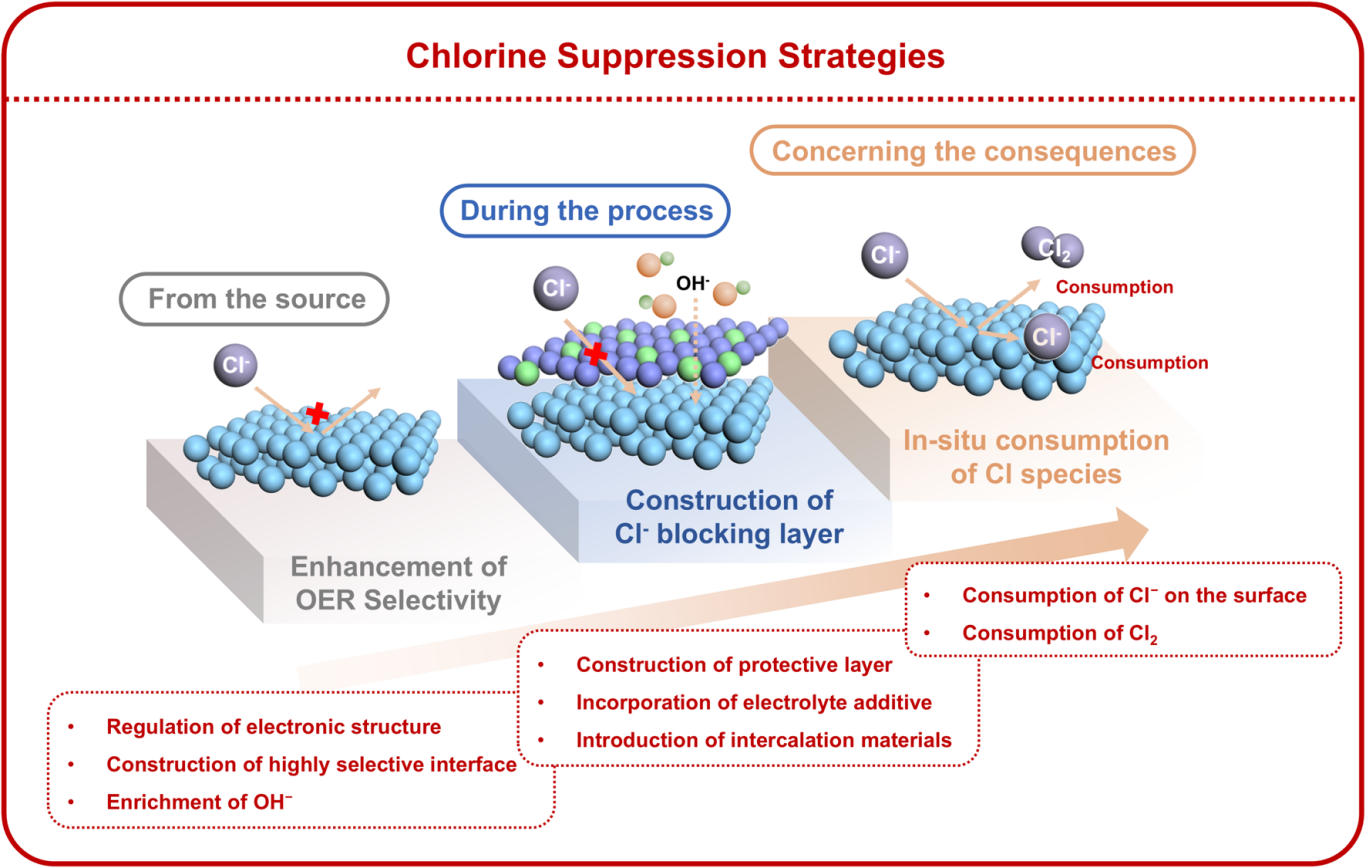
Three key strategies for catalyst design for chlorine suppression at different reaction stages

This review summarized three widely adopted catalyst design strategies to suppress the ClER, as illustrated in Fig. 3, (1) enhancement of OER selectivity: the catalytic activity toward the OER can be optimized through the regulation of electronic structure, construction of a highly selective interface, and enrichment of OH⁻, and the occurrence of ClER could be minimized; (2) construction of Cl^−^ blocking layer: the likelihood of ClER is significantly reduced when the contact between Cl^−^ ions and active sites is inhibited, which can be achieved through the construction of a protective layer, the incorporation of electrolyte additives, and the introduction of intercalation materials; (3) in situ consumption of chlorine species: this approach prevents the accumulation of chlorine species (Cl^−^ and Cl_2_) on the electrode surface, thereby mitigating continuous catalyst degradation.

**Fig. 4:**
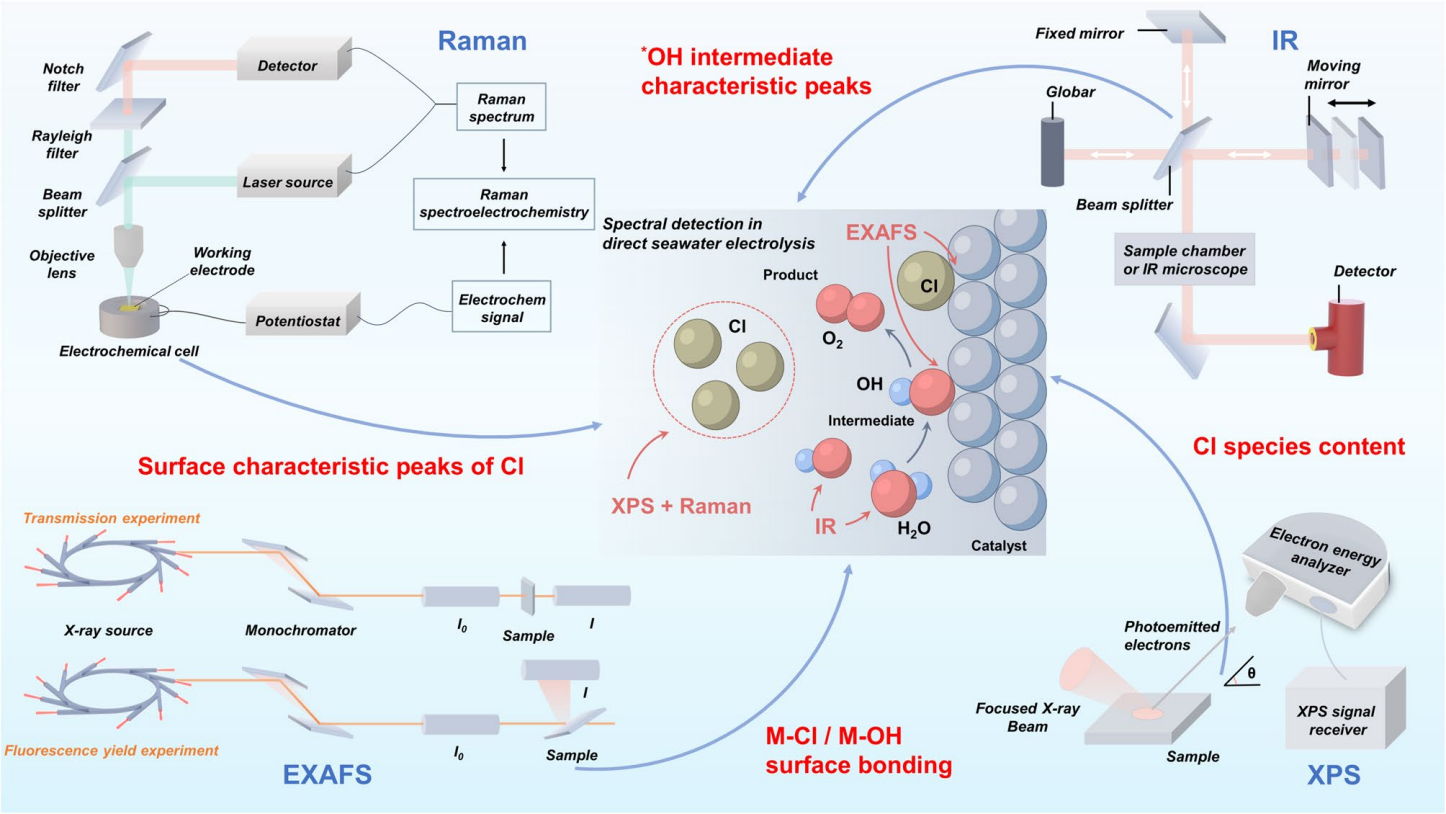
Spectroscopic techniques for detecting chlorine species in direct seawater electrolysis

The detection of Cl^−^ and other intermediates is crucial for the effective implementation of chlorine suppression strategies. Spectroscopic techniques play a pivotal role in elucidating these intermediates in the seawater electrolysis process [80]. Figure 4 presents an overview of four spectroscopic methods, including Raman and in situ infrared (IR) spectroscopy, employed to identify Cl^−^ and related intermediates, as well as to characterize their bonding states with precision. Zhou et al. employed in situ Raman spectroscopy to detect the presence of Cl^−^ on the material surface before reconstruction. Notably, the characteristic Cl^−^ peak disappeared after reconstruction, indicating that the reconstructed NiFeCo(OH) compound exhibits a chlorine-repellent property [81]. Similarly, X-ray photoelectron spectroscopy (XPS) was employed by Qiao et al. to investigate the Cl^−^ species on the surface of electrode material, thereby exploring its OER selectivity [82]. In situ IR was employed to detect the ^*^OH intermediate during the reaction process, further enabling the detection of local pH changes [83]. The substantial generation of ^*^OH on the material surface significantly inhibited Cl^−^ adsorption and promoted the OER by strengthening the M-OH bond. Fourier transform extended X-ray absorption fine structure (EXAFS) enables precise observation of the adsorption states of intermediates during direct seawater electrolysis. By comparing the bond strengths between active sites and Cl^−^ or OH^−^, the catalytic chlorine suppression effect of the electrode can be predicted [84].

### Enhancement of OER Selectivity

In direct seawater electrolysis, the OER and ClER are severely competing due to the high concentration of Cl^−^ ions. The catalysis primarily acted as the bridge of electron transfer connecting adsorbates and active sites. How to ensure the massive engagement of active sites selectively catalyzing the OER pathway rather than ClER at the given potential is the key for Cl_2_ reduction. The superior OER selectivity allowed a desired high current density achieved at a lower overpotential, effectively suppressing the ClER and reducing energy consumption of the electrolysis [85]. In this section, we outlined three key strategies for superior OER catalyst design: electronic structures regulation, high-selectivity interfaces construction, and local OH^−^ concentration enrichment.

**Fig. 5:**
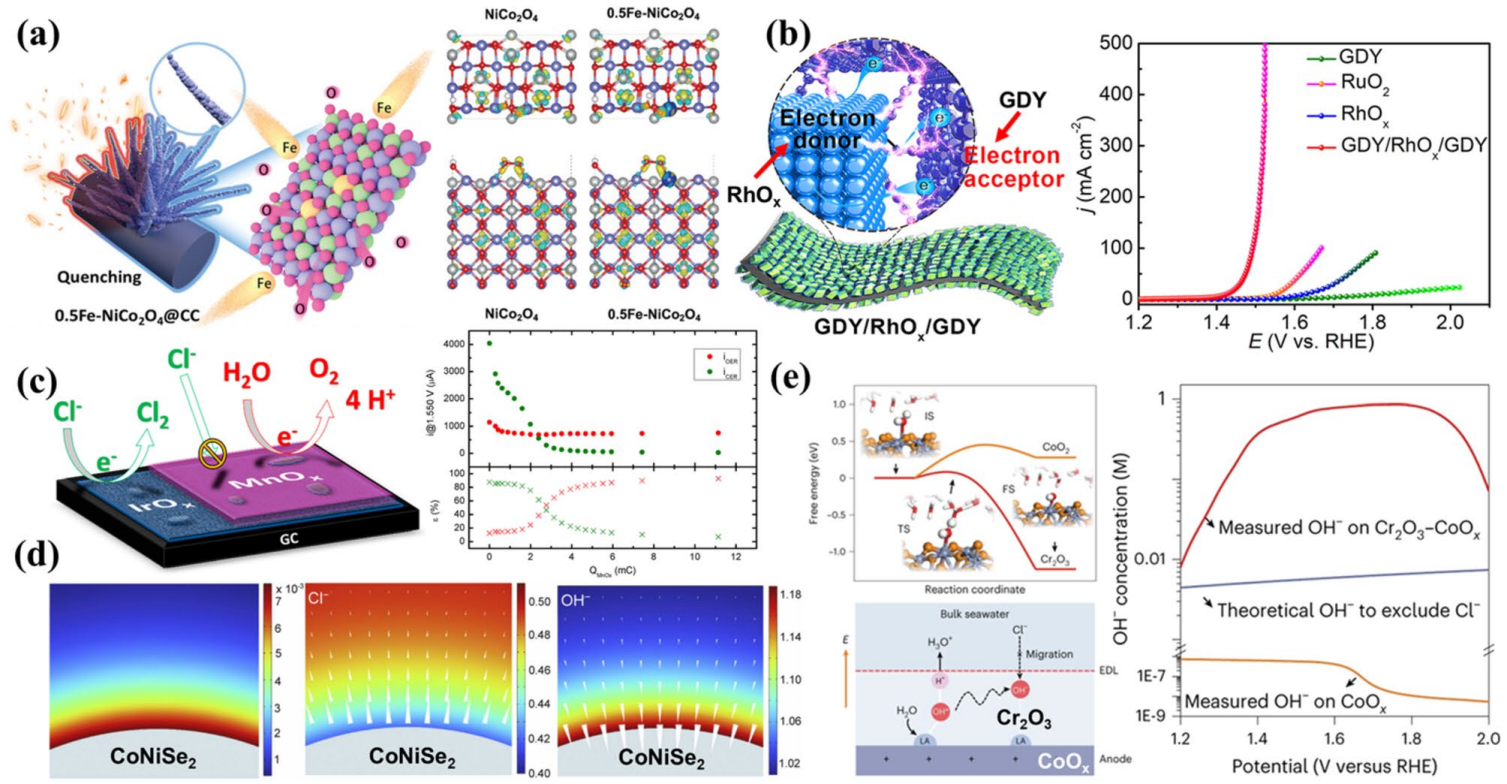
**a** Modulation of the electronic structure of porous NiCo_2_O_4_ nanowires by Fe doping [93]. **b** Construction of GDY/RhO_x_/GDY heterostructures to provide highly selective interfaces [99]. **c** Introduction of MnO_x_ interfaces to improve OER selectivity [101]. **d** Finite element simulations of OH^−^ enrichment by SeO_4_^2−^ space-charged layers [104]. **e** Cr_2_O_3_ modulation of localized microenvironment for seawater electrolysis [82]

The catalysts electronic structure was served as the bridge between material structure and its catalytic functionality, highly influential to absorbent adsorption, activation, and downstream conversion [86–89]. By modulating the electronic structure of the catalyst, it is possible to optimize reactant adsorption strength and effectively lower the energy barrier for RDS from kinetic perspective [90]. The increased electron density for active sites promotes the activation of reactant molecules through strong electron coupling effects [91]. This electronic structure promotes charge transfer between the catalyst and reactants, thus promoting efficient reactant activation [92]. The synergistic effect of these improvements, which enables optimal adsorption of reactants and their activation, significantly enhances the catalytic efficiency and selectivity. Pan et al. prepared porous NiCo_2_O_4_ nanowires with oxygen vacancies and surface-doped Fe atom using a rapid quenching method [93]. This method, which effectively modulated the electronic structure and surface properties of the catalyst, introduced a high concentration of oxygen vacancies, providing more active sites for the OER process. The Fe-doped catalyst exhibited strong electronic coupling effects (Fig. 5a), highlighting the critical role of Fe doping in modulating the electronic state and structure. The catalyst demonstrated similar overpotential of 258 mV in freshwater and 293 mV in seawater at a current density of 10 mA cm^−2^, indicating that Fe doping effectively suppressed the ClER during seawater electrolysis by altering the electronic structure. The Cr is regarded as an ideal corrosion inhibitor in the seawater due to its unique electronic configuration (t_3_^2^g e_0_g) [94]. Leveraging this property, Huang et al. proposed a work function engineering strategy by doping vein-like Cr into Co_x_P, achieving electronic coupling and charge density redistribution [95]. This doping facilitated efficient electron transfer between Cr-Co_x_P and adsorbed oxygen, effectively lowering the energy barrier of the rate-determining step of the OER. When applied to seawater electrolysis, this catalyst achieved nearly 100% FE for both HER and OER, with no hypochlorite detected in the electrolyte, demonstrating its excellent OER selectivity.

The adsorption and desorption of reactants can be precisely controlled by highly selective interfaces, which are engineered through tailored surface structures and the chemical compositions of the catalyst. These interfaces selectively facilitate desired reaction pathways while inhibiting side reactions [96, 97]. By constructing such interfaces, the spatial distribution and density of active sites can be finely regulated, ensuring efficient interaction between reactants and catalytic sites [98]. For instance, Li et al. developed a bilayer heterostructure of graphdiyne/RhO_x_/graphdiyne (GDY/RhO_x_/GDY) on RhO_x_ nanocrystals [99]. This heterostructure created *sp*-hybrid Carbon–Oxygen–Rhodium bilayer intercalation interfaces, which provided abundant active sites at the *sp*-hybrid Carbon–Oxygen–Rhodium (*sp*-C ~ O-Rh) junctions. As depicted in Fig. 5b, these interfaces exhibited superior OER catalytic activity in seawater electrolysis. Similarly, MnO_x_, a non-precious metal-based catalyst, demonstrated excellent OER selectivity under acidic conditions and is among the few catalysts capable of maintaining moderate stability in such environments [100]. Based on this, Koper et al. [101] deposited MnO_x_ onto IrO_x_ and evaluated the catalytic behavior of this interface. As shown in Fig. 5c, the three-electrode system ClER selectivity dramatically decreased from 86% to less than 7% in the presence of 30 mM Cl^−^, highlighting that the introduction of MnO_x_ significantly enhanced OER selectivity.

The OER selectivity enhancement can also be achieved through the enrichment of OH^−^ at the surface of the catalyst. The OER typically follows a multi-step proton-coupled electron transfer mechanism, while ClER involves the oxidation of Cl^−^. The presence of OH^−^ stabilizes OER intermediates and facilitates the preferred OER pathway [102]. Moreover, by increasing the concentration of OH^−^, the availability of Cl^−^ is decreased, which in turn lowers the energy barrier for the OER reaction and makes it more thermodynamically favorable [103]. Wang et al. demonstrated this principle using a novel CoNiSe_2_ catalyst, incorporating a SeO_4_^2−^ space-charge layer. As shown in Fig. 5d, finite element simulations indicated that OH^−^ concentration on the catalyst surface exceeded that of Cl^−^, suggesting selective OH^−^ enrichment [104]. This high concentration of surface OH^−^ reduced energy barriers for deselenization and dehydrogenation, facilitating rapid catalyst reconstruction and the formation of highly active Co-NiOOH, which significantly boosted OER performance. Ling et al. [82] proposed an alternative approach to OH^−^ enrichment by incorporating a Lewis acid layer on the catalyst surface, promoting water molecule dissociation and capturing in situ generated OH^−^ (Fig. 5e). This localized alkalinity effectively inhibited chlorination on the surface of catalyst. Additionally, the introduction of Cr_2_O_3_ created an acidic microenvironment that enriched OH^−^, thermodynamically favoring the OER. As demonstrated through density functional theory (DFT) simulations (Fig. 5e), the energy barrier for water dissociation of Cr_2_O_3_ was significantly lower than that of CoO under identical potential conditions.

The OER selectivity enhancement of catalysts is an effective approach to suppress the ClER, serving as a robust chlorine suppression strategy. However, it is insufficient to rely solely on the intrinsic selectivity of active sites, as Cl^−^ intrusion can irreversibly deactivate these sites, leading to significant economic losses in industrial-scale water electrolysis. Thus, it is imperative to develop novel strategies that prevent Cl^−^ from interacting with active sites to mitigate catalyst degradation and ensure sustained operational efficiency.

### Construction of Cl^−^ Blocking Layer

Construction of Cl^−^ blocking layer on the electrode surface is another effective strategy to suppress the ClER. This inhibition mitigates corrosion and reduces the incidence of the ClER, thereby promoting the OER and enhancing overall electrolysis efficiency [70, 105]. Furthermore, this barrier layer can decelerate the degradation of catalytic active sites, facilitating the long-term stable operation of the electrolysis system at elevated current densities. Subsequently, three primary methods for constructing the Cl^−^ blocking layer are investigated: the development of protective layers, the adoption of electrolyte additives, and the introduction of specialized insertion materials.

**Fig. 6:**
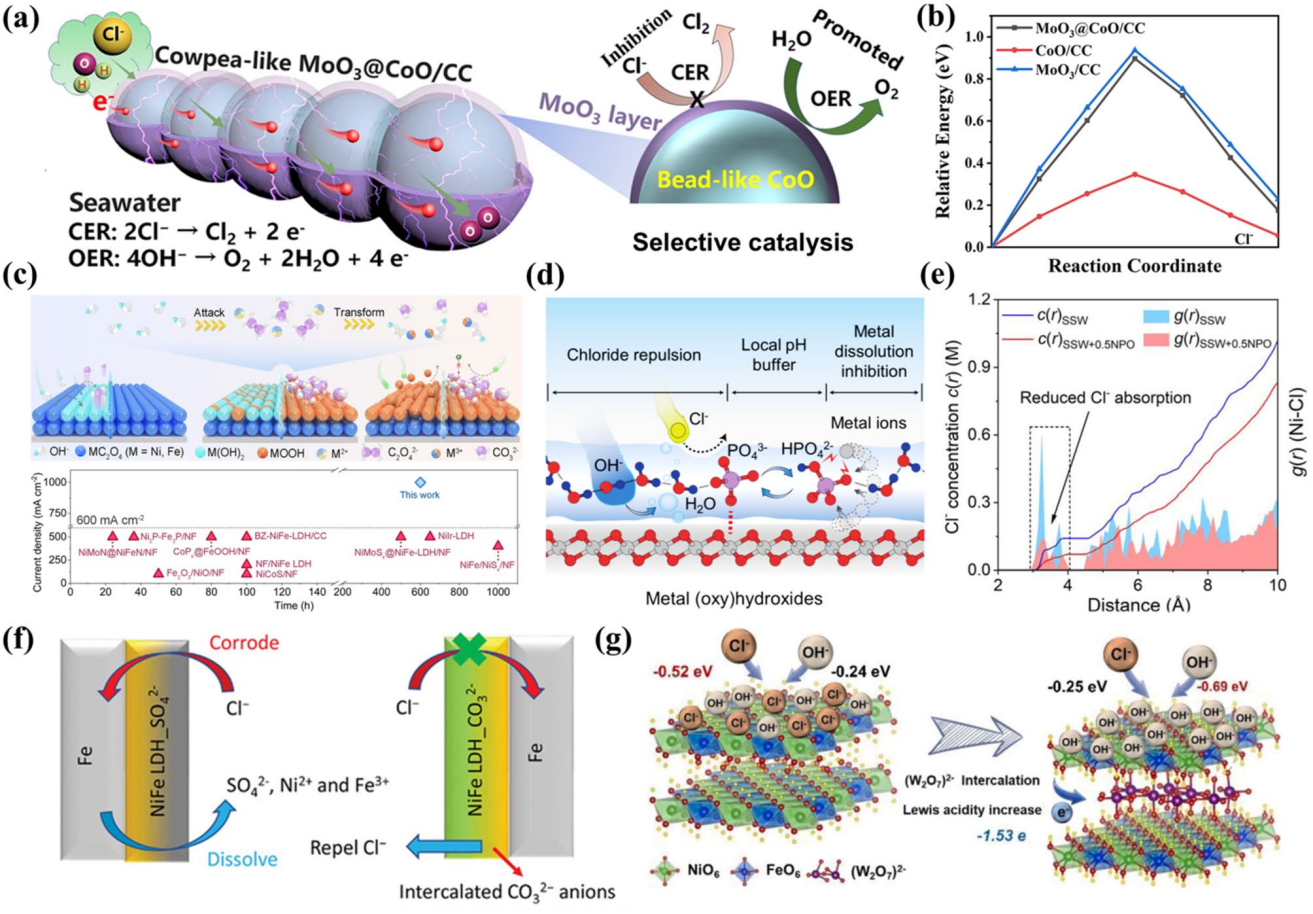
**a** Schematic diagram of Cl^−^ transfer blocking by MoO_3_ protective layer [108]. **b** Reaction energy barriers of Cl^−^ in the catalyst before and after the introduction of MoO_3_ protective layer [108]. **c** Mechanism of in situ carbon–oxygen anion self-transformation for the transformation of NiFe oxalate to carbonate [12]. **d** Mechanism of blocking of Cl^−^ transfer by Na_3_PO_4_ additives [112]. **e** Radial distribution function g(r) of Ni-Cl and the chloride ion concentration function c(r) as a function of the distance from the electrode, with and without the addition of 0.5 mol Na_3_PO_4_ [112]. **f** NiFe-LDH introduces CO_3_^2−^ intercalation to block Cl^−^ transfer [119]. **g** Schematic representation of HSAB principle and adsorption energies for OH^−^ and Cl^−^ [121]

In seawater electrolysis, catalyst reconstruction is a prominent phenomenon, and understanding the true catalytic active sites is essential for comprehending the catalytic mechanism [106]. Deng et al. have extensively investigated the reconstruction behavior of the NiMoFe/NM catalyst during seawater electrolysis using in situ Raman spectroscopy [107]. At 1.34 V, the characteristic peaks of α-Ni(OH)_2_ in the Raman spectrum disappear, replaced by new peaks at 476 and 554 cm^−1^, corresponding to the *E*_g_ bending vibrations and A_1g_ stretching vibrations of NiIII-O in γ-NiOOH, respectively. As the voltage increases to 1.52 V, the intensity of the γ-NiOOH peaks strengthens, signifying the catalyst surface has stabilized, and the OER progresses steadily. Upon reducing the voltage to 1.23 V, the γ-NiOOH peaks revert to α-Ni(OH)_2_ within 2 h, confirming the dynamic reversibility of the active species. This catalyst reconstruction not only highlights its structural evolution during the OER but also offers valuable insights into mitigating ClER in seawater electrolysis. Wang et al. employed atomic layer deposition (ALD) to incorporate an ultra-thin amorphous MoO_3_ layer into a bead-like CoO array systematically arranged on a three-dimensional carbon cloth, thereby creating a catalyst with a cowpea-like architecture. The structural schematic is illustrated in Fig. 6a [108]. As shown in Fig. 6a, the precise modulation of CoO surfaces with MoO_3_ effectively reduces the overpotential and enhances the interfacial reactivity. This modulation allows for precise control over the formation of *O and *OOH. It optimizes reaction pathways and accelerates the kinetics of the OER. Additionally, the MoO_3_ layer serves as an effective barrier against Cl^−^ ion penetration at the catalytic interface, and the stable, reconstructed CoMo-LDH layer provides further chloride ion rejection through electrostatic repulsion, thus enabling selective oxidation in the seawater. DFT simulations were conducted to assess the migration energy barrier of Cl^−^ within the catalyst before and after the introduction of the MoO_3_ layer, as shown in Fig. 6b. The results indicated that the MoO_3_ layer effectively obstructs Cl^−^ from reaching the catalytically active interface. Furthermore, the stable CoMo-layered double hydroxide (LDH) formed via the reconstruction of the MoO_3_ layer repels Cl^−^ ions through electrostatic interactions, thereby significantly mitigating corrosion and the ClER. Apart from MoO_3_, the use of other physical barrier layers can also effectively suppress the influence of chlorine. In the study by Yang et al., V_2_O_3_ was coupled with Pt-Ni_3_N. The V_2_O_3_ layer, due to its Lewis acid properties, adsorbs excess OH^−^ ions. This creates a local, highly alkaline microenvironment on the electrocatalyst surface [109]. The V_2_O_3_ protective layer not only reduces Cl^−^-induced corrosion of catalytic active sites but also limits the interaction between metal cations (e.g., Ca^2+^ and Mg^2+^) and OH^−^ in seawater. This helps to decrease the formation of insoluble precipitates. Hao et al. constructed a protective layer composed of MoO_x_ and PO_x_ on the surface of Mo-NiP@NF electrodes using a mild electroless plating technique [110]. The coexistence of PO_x_^δ−^ and MoO_x_^δ−^ ions on the electrode surface generates an electrostatic repulsion effect, effectively protecting the electrode material from corrosion by Cl^−^. This ensures the stability and durability of the Mo-NiP@NF electrodes in harsh marine environments. In addition to directly constructing a protective layer during synthesis, in situ transformation to generate a protective layer represents a viable strategy for forming the Cl^−^ barrier. This method enables dynamic barrier formation during the reaction process, adapting to fluctuating conditions and thereby more effectively inhibiting chloride ion contact with the active sites. Tang et al. reported an in situ carbon–oxygen anion autoconversion mechanism that transformed Nickel–Iron oxalate (NiFe-C_2_O_4_) into carbonate [12]. This spontaneous and efficient transformation effectively shielded catalyst active sites from Cl^−^ erosion. During this conversion, NiFe-C_2_O_4_ reorganized into a relatively stable configuration, and the released CO_3_^2−^ ions repel Cl^−^ and promoted the formation of high valence state for active sites. This mechanism not only enhances the OER activity of the catalyst but also substantially improves its stability. Notably, its performance in the relevant literature is commendably high, as presented in Fig. 6c.

The incorporation of electrolyte additives in direct seawater electrolysis could mitigate corrosion against ClER. These additives either form stable complexes with Cl^−^ or generate a protective layer on the electrode surface via physical and chemical interactions, thereby effectively preventing Cl^−^ from accessing catalytically active sites. Additionally, certain additives can enhance the electrochemical stability of the electrolyte and minimize side reactions, thus improving overall electrolysis efficiency and stability [111]. Among these additives, phosphate ions (PO_4_^3−^) exhibit high electrochemical stability and substantial electrostatic potential, allowing them to interact with water through hydrogen bonding to create a “semipermeable layer” on the surface of the electrode [112]. This layer effectively repelled Cl^−^ while minimally impeded the diffusion of OH^−^. The abundant hydrogen bonding between PO_4_^3−^ and water molecules facilitates OH⁻ transport, enabling easier diffusion through the semipermeable layer formed by PO_4_^3−^. In contrast, the Cl^−^ is a weak hydrogen-bonding acceptor, and the Coulomb repulsion between PO_4_^3−^ and Cl^−^ predominates leaded to effective inhibition of Cl^−^ within the layer, as illustrated in Fig. 6d [112]. The addition of Na_3_PO_4_ to the electrolyte significantly reduces Cl^−^ concentrations of approximately 50% within the thicknesses of 4 Å and of approximately 30% for thicknesses of 10 Å, as shown in Fig. 6e. It supported the effectiveness of PO_4_^3−^ in diminishing Cl^−^ adsorption and alleviating electrode corrosion. Similarly, the inclusion of sulfate ions (SO_4_^2−^) in the electrolyte effectively mitigates the corrosive impact of Cl^−^ at the anode. When sulfate was employed as an additive, SO_4_^2−^ preferentially adsorbed onto the anode surface, forming a negatively charged protective layer. This negatively charged layer induced electrostatic repulsion force to prevent Cl^−^ from the anode, thereby significantly enhancing corrosion resistance [113]. Furthermore, due to the ideal ion potential of CrO_4_^2−^, its addition as an additive can significantly repel Cl^−^ ions near the catalyst, thereby enhancing the corrosion resistance of the catalyst [114]. Cr^6+^ has fully unoccupied d-orbitals and can accept electrons from Ni atoms, which improves the catalytic performance for OER. The introduction of CrO_4_^2−^ simultaneously enhances the activity and stability of electrode materials in the seawater electrolysis process.

The Nickel–Iron-layered double hydroxides (NiFe-LDHs) have demonstrated significant OER activity in the freshwater electrolysis [115]. However, pure LDH encounter challenges such as high onset potentials, limited intrinsic conductivity, and weak OH^−^ selective adsorption capacity. These limitations could bring a high overpotential under high current density conditions [116, 117], necessitating improvements in corrosion resistance [118]. To address these issues, the introduction of intercalation materials could modify the surface properties of the catalyst and facilitates the formation of a protective layer against Cl^−^ interaction with active sites. Lu et al. successfully synthesized a cost-effective and scalable carbonate-intercalated NiFe-LDH catalyst through etching hydrolysis and ion exchange [119]. The incorporation of carbonates effectively diminished Cl^−^ adsorption on the catalyst surface, preventing the interaction between metal atoms and Cl^−^ ions, thereby suppressing the corrosive effects of Cl^−^ on the anode and significantly improving catalytic stability, as illustrated in Fig. 6f. According to Pearson’s Hard and Soft Acids and Bases (HSAB) principle, harder acids preferentially bind to harder bases [120]. Zhou et al. introduced the (W_2_O_7_)^2−^ anion as an intercalation layer in NiFe-LDH to regulate the oxidation states of nickel and iron. The incorporation of W^6+^ increased the Lewis acidity of NiFe-LDH, which subsequently favored binding to OH^−^, thereby forming a barrier that inhibits Cl^−^ transfer. As shown in Fig. 6g, this NiFe-LDH with (W_2_O_7_)^2−^ intercalation exhibited exceptional resistance to chlorine interference and demonstrated enhanced corrosion resistance due to its OH^−^ barrier structure [121]. Additionally, owing to their high charge density, small size, and unique trigonal planar structure, CO_3_^2−^ establishes strong electrostatic interactions with the positively charged host layers of LDH. By intercalating CO_3_^2−^ into the CoFe-C_i_ nanosheets, Feng et al. not only enhanced the structural stability but also reduced the interlayer spacing [122]. This narrowing of the interlayer effectively prevents Cl^−^ ions from displacing carbonate ions through cation exchange, thereby preserving the layered structure during seawater electrolysis and significantly improving the stress resistance of the electrocatalyst.

The introduction of chlorine suppression protective layers has become a prominent area of research in direct seawater electrolysis, providing local protection against the corrosion of catalytic active sites by Cl^−^. However, the omnipresence of Cl^−^ in the seawater leads to the inevitability of instances where it can inflict irreversible damage on the equipment. Therefore, it warrants thoughtful consideration to address the challenge of unimpeded Cl^−^ during seawater electrolysis and to explore methods for integrating the chlorine chemistry of seawater with other industrial processes to generate high-value-added products.

### In Situ Consumption of Chlorine Species

Although various strategies have been implemented to enhance the OER selectivity and establish Cl^−^ blocking layers to mitigate ClER, these approaches are not entirely effective in preventing the ClER. During seawater electrolysis, the Cl^−^ can still infiltrate the catalyst layer, leading to direct contact with the metal bipolar plate, and subsequently bring the corrosion [123, 124]. Furthermore, while the amount of Cl_2_ is relatively low, its corrosive impact on industrial-scale equipment of seawater electrolysis over extended periods is significant and cannot be ignored [125, 126]. Consequently, the in situ consumption of chlorine species (Cl^−^ or Cl_2_) during direct seawater electrolysis is a critical challenge that warrants further investigation and targeted research.

**Fig. 7:**
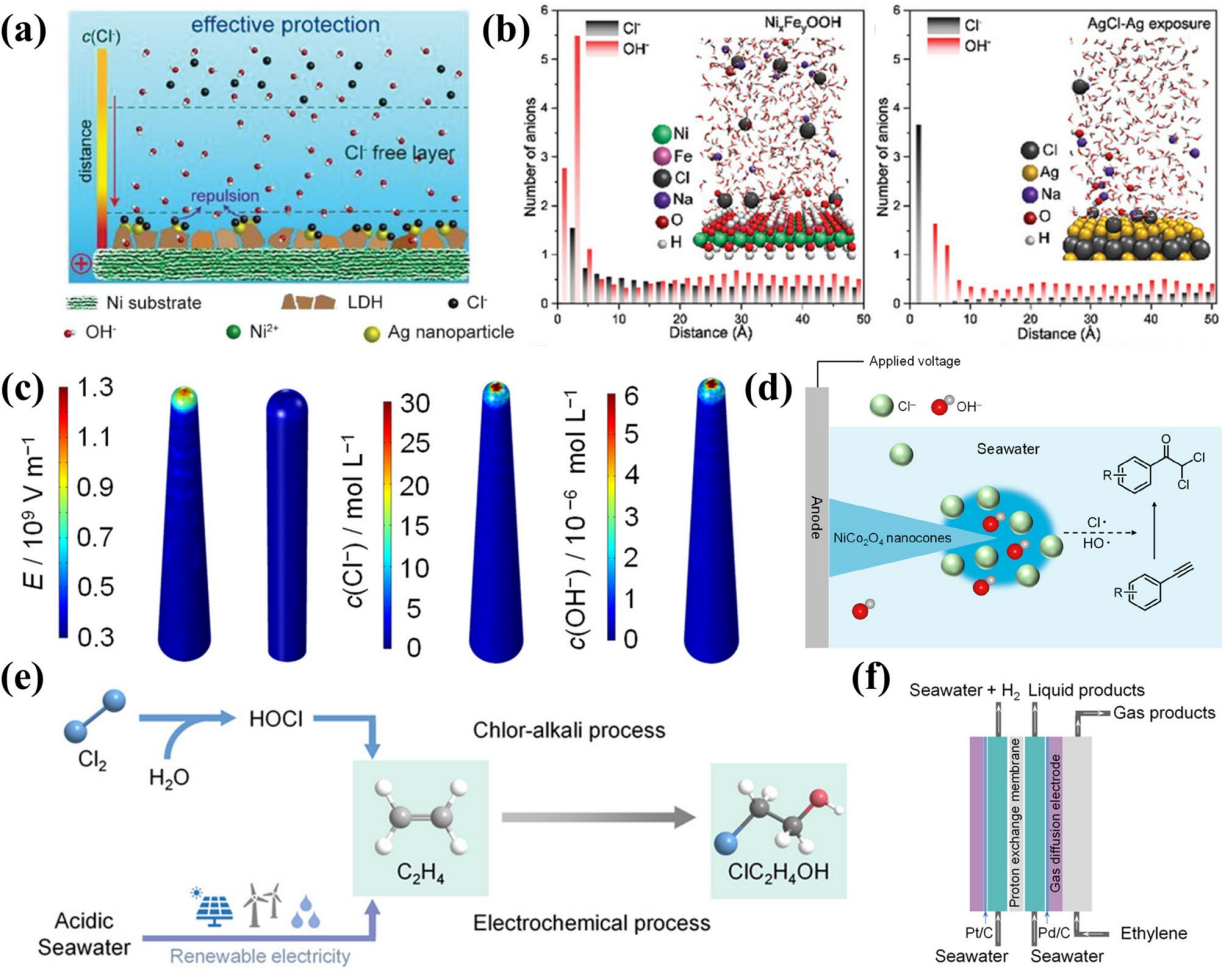
**a** Schematic representation of the effect of chloride ion immobilization strategies on chloride ion corrosion protection [127]. **b** MD simulation of the amount of Cl^−^ and OH^−^ versus the distance between the exposed surfaces of Ni_x_Fe_y_OOH and AgCl-Cl [127]. **c** Finite element simulation of the electric field and the distribution of Cl^−^ and OH^−^. over the surface of the catalysts [128]. **d** Schematic representation of the electrolysis of seawater for the synthesis of α,α -dichloroketones by electrolysis of seawater [128]. **e** Schematic diagram of the current chlor-alkali process and the electrochemical process of ethylene to dichloroethanol [130]. **f** Schematic diagram of a proton exchange membrane electrolyzer [130]

To address this issue, Lu et al. [127] proposed a Cl^−^ immobilization strategy by uniformly integrating Ag nanoparticles into the NiFe-LDH catalysts surface. The embedded Ag reacted with free Cl^−^ to form insoluble AgCl, effectively immobilizing the Cl^−^ ions. This approach reduces the amount of free Cl^−^ species and also repels remaining Cl^−^ ions through strong co-ionic repulsion between surface chlorine atoms on AgCl and free Cl^−^, as illustrated in Fig. 7a. Figure 7b describes the molecular dynamics (MD) simulations of Ni_x_Fe_y_OOH interactions with AgCl, revealing Cl^−^ initially drawn toward the Ni_x_Fe_y_OOH surface driven by electrostatic forces. However, exposed chlorine atoms on AgCl exert strong repulsion on Cl^−^ anions, particularly those within 3 Å of the surface. This in situ immobilization and repulsion strategy significantly enhanced the corrosion resistance. It achieved significant improvement with stable operation for over 5000 h at a current density of 400 mA cm^−2^ for advancing seawater electrolysis technology. In addition to immobilizing Cl^−^, direct consumption of Cl^−^ is another effective approach to mitigate its corrosive effects. Wu et al. developed the NiCo_2_O_4_ nanocones with high curvature, which effectively enrich the concentration of OH^−^ and Cl^−^ ions from seawater [128]. These ions could serve as feedstocks for synthesizing α,α-dichloroketones, with the nanocone ion enrichment effect validated by finite element simulations (Fig. 7c). In this process, the Cl^−^ undergoes electrooxidation to form the Cl· radicals; then, it attacked the α-carbon of alkynes to generate vinyl radicals, followed by further transformations as shown in Fig. 7d. This method reduces Cl^−^-induced corrosion and also generates high-value-added pharmaceutical products.

Beyond mitigating the corrosion of the electrolyzer by consuming Cl^−^, the Cl_2_ produced during ClER also poses a significant corrosion risk. As the formation of Cl_2_ is unavoidable, developing strategies for its in situ depletion to minimize corrosive effects is a critical research focus. While the Cl_2_ is often considered an undesirable by-product in the seawater electrolysis for hydrogen and oxygen generation, it has a pivotal role in chlorohydrin synthesis technology [78, 129]. Specifically, the gaseous Cl_2_ could react with water into HClO and it could break carbon–carbon double bonds of ethylene and transform into 2-chloroethanol, as illustrated in Fig. 7e. Qiao et al. leveraged this process by integrating seawater electrolysis with the electro-oxidation of ethylene, achieving a FE of 68% for 2-chloroethanol production [130]. This approach generated H_2_ under acidic conditions and also produced high-value 2-chloroethanol (Fig. 7f). This work offers a pioneering idea for in situ Cl_2_ consumption strategies to mitigate the corrosion of electrolyzer. Similarly, Sun et al. demonstrated that Cl_2_ generated at the anode can be transformed in situ into HCl, chlorinated polymers, and precursors for bleaching agents. At the same time, the elevated local pH during hydrogen production at the cathode facilitates CO_2_ fixation [131]. This idea promoted Cl_2_ in situ consumption and simultaneously integrated hydrogen production and CO_2_ fixation, aligning with current initiatives toward “carbon neutrality” and “carbon peaking” goals [132].

The in situ consumption of chlorine species plays a pivotal role in mitigating chloride-induced chemical corrosion. Moreover, by integrating this process with other industrial reactions, it facilitates the generation of higher-value products such as alcohols and ketones. This idea could drive the transformation of harmful chlorine species into a beneficial outcome with enhanced overall efficiency and derived high-value-added products.

To address the challenge of the ClER in direct seawater electrolysis, recent researches focused on developing catalyst design for higher FE and suppress the Cl_2_ formation. There have been promoted strategies including electronic structures regulation, interface engineering, local OH^−^ concentration adjustment, and protective layers construction against ClER. However, complete ClER suppression still remains challenging, emphasizing the need for in situ chlorine depletion to reduce corrosion and convert chlorine species into value-added products. The chlorine suppression strategy necessitates a profound comprehension of the underlying scientific principles governing existing materials and their interfacial dynamics. Concurrently, attention could be paid into the development of advanced system architectures for optimized Cl^−^ management and utilization, which is another effective pathway to well optimize the efficiency, stability, and economic viability of direct seawater electrolysis.

## Seawater Electrolysis Systems

The advancements in seawater electrolysis catalysts highlight hydrogen energy potential as a sustainable alternative to fossil fuels. Beyond the critical need to develop catalysts that mitigate the ClER, other cations and impurities present further obstacles to the electrolysis process. Consequently, the development of seawater electrolysis systems has emerged as a prominent focus. The anion exchange membrane (AEM) electrolysis system exhibits significant potential for seawater electrolysis. It could operate in alkaline conditions and utilize cheaper non-precious metal catalysts and hold potentials for seamlessly integrating with renewable energy sources. This section highlights low-temperature, high-efficiency AEM seawater electrolysis systems and further summarizes emerging technologies over the past five years. The designing principles of these advanced systems, emphasizing their pivotal roles and advantages in direct seawater electrolysis. This review aims at offering insights to advance the development and application of direct seawater electrolysis technologies.

### Anion Exchange Membrane Electrolyzer

As an emerging technology, the AEM electrolyzer offers a promising pathway for advancing seawater electrolysis systems [11, 133]. Similar to traditional electrolyzers function, its mechanism involves the reduction of water molecules at the cathode, leading to the production of the H_2_ and the OH^−^ ions (Eq. 18). The OH^−^ ions are transported through the AEM to the anode, where they participate in an oxidation reaction to generate the O_2_ (Eq. 19).

18 $$2\hbox{H}_{2} \hbox{O}{\text{ + 2e}}^{ - } \to \hbox{H}_{2} + 2\hbox{OH}^{ - }$$

19 $$4\hbox{OH}^{ - } -{\text{ 4e}}^{ - } \to \hbox{O}_{2} + 2\hbox{H}_{2} \hbox{O}$$

**Fig. 8:**
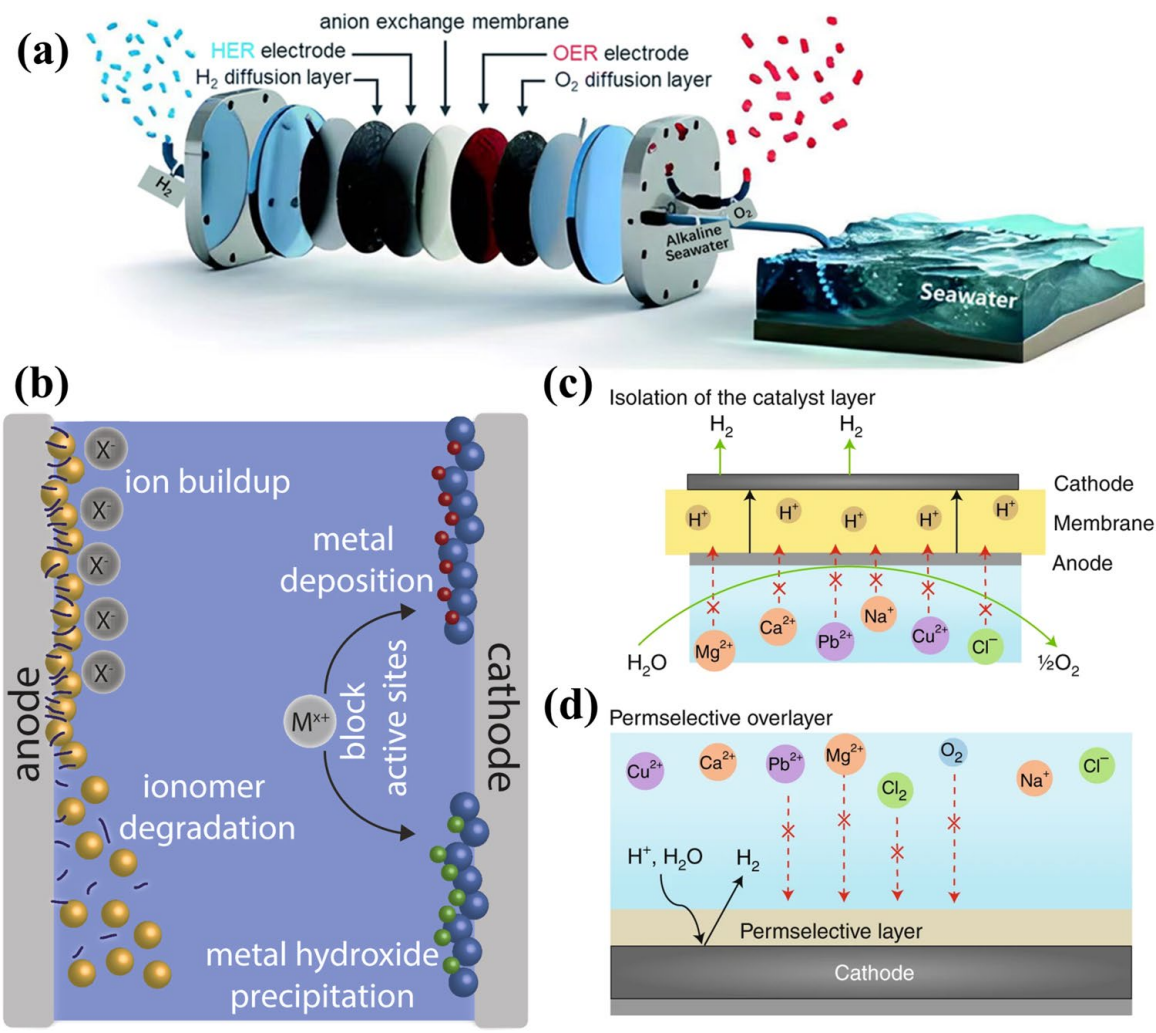
**a** Schematic diagram of hydrogen production in an AEM water electrolyzer [134]. **b** Possible degradation mechanism of the electrolyzer caused by impurities [156]. **c** Inhibition of catalyst deactivation by highly selective permeable membranes [77]. **d** Construction of a selective protective layer blocking the contact of impurities with the catalyst [77]

The AEM electrolyzer operates in an alkaline environment at the membrane interface, typically operating at low-concentration alkaline electrolytes. The seawater electrolysis mechanism is illustrated in Fig. 8a [134]. However, unlike freshwater electrolysis, seawater electrolysis presents extra challenges for AEM electrolyzers due to the high Cl^−^ content, which poses significant corrosion risks. To mitigate this, the design of AEM electrolyzers prioritizes the optimization of OH^−^ transport by employing electrode materials with high anionic and electronic conductivity, adjusting the operating conditions (e.g., maintaining an alkaline environment), and improving the structure of the AEM, thereby minimizing the Cl^−^ and other cations exchanging with OH^−^. This targeted approach effectively restricts Cl^−^ permeation through the membrane, enhancing the resistance of the electrolyzer to Cl^−^-induced corrosion, particularly in high-salinity environments, thus improving process stability and longevity [135].

In contrast, the proton exchange membrane (PEM) electrolyzers [136, 137], which conduct protons, are more vulnerable to corrosion in chloride-rich environments due to potential Cl^−^ infiltration that compromises membrane integrity [138]. The PEM electrolyzers operating under acidic conditions require noble metal catalysts, which significantly increase operational costs [139–141]. The alkaline electrolyzers, on the other hand, are well-established and widely utilized due to their cost-effective nature compared to the PEM systems. They support OER in alkaline environments by leveraging the greater potential difference between OER and ClER at elevated pH levels, thereby enhancing the OER selectivity [142, 143]. However, their reliance on concentrated alkaline solutions accelerates the corrosion of the equipment, especially during start-stop cycles, and presents integration challenges with intermittent renewable energy sources [144].

Similarly operating in alkaline conditions, the AEM electrolyzers exhibit enhanced OER selectivity during seawater electrolysis, as the AEM selectively conducts the OH⁻ ions, preventing the passage of other ions [145]. Moreover, they exhibit rapid responsiveness to fluctuating input power, quickly modulating the electrolysis rate to adapt to the intermittent and variable nature of renewable energy sources such as wind and solar power [146]. The alkaline environment enables the use of cost-effective, non-precious metal catalysts, distinguishing AEM electrolyzers from PEM electrolyzers [147–149]. This adaptability allows AEM electrolyzers to efficiently scale up under dynamic energy inputs, maintaining high stability and energy conversion efficiency. Specifically, AEM electrolyzers can achieve nearly 100% Faradaic efficiency with non-precious metal catalysts [150], which is comparable to or even surpasses the performance of PEM electrolyzers that rely on more expensive precious metal catalysts. Moreover, studies have shown that certain non-precious metal catalysts in AEM electrolyzers demonstrate superior stability, maintaining over 95% of their initial activity after continuous operation for over 5000 h [151], whereas PEM electrolyzers may experience significant performance degradation due to corrosion under similar conditions [152]. Additionally, AEM electrolyzers exhibit 10–15% higher energy conversion efficiency compared to PEM electrolyzers, which translates to reduced energy consumption and lower operational costs in practical applications [153]. These advantages make AEM electrolyzers a more sustainable and economically viable option for large-scale hydrogen production from seawater. Their reduced start-stop losses minimize mechanical stress and system wear during frequent adjustments, translating into lower maintenance costs [154]. Additionally, the utilization of non-precious metal catalysts further decreases operational expenses. These attributes enable AEM electrolyzers to enhance energy efficiency and significantly lower operating costs when integrated with renewable energy systems, highlighting their potential for sustainable energy conversion. Wang et al. indicates that, compared to traditional alkaline water electrolysis, integrating AEM electrolyzers with renewable energy systems can reduce energy consumption by 40%–50% [68]. This enhancement in energy efficiency is primarily due to the effective control of overpotentials for hydrogen and oxygen during electrolysis. The baseline levelized cost of hydrogen (LCOH) for AEM electrolyzers is estimated to be $5.79 per kilogram, with an optimal current density of 1.38 A cm^−2^, balancing stability and performance to achieve the lowest LCOH [155]. Furthermore, Lu et al. employed a NiFeBa-LDH catalyst for AEM seawater electrolysis, achieving an OER selectivity exceeding 99% in alkaline saline solutions. Under simulated industrial conditions, the electrolyzer demonstrated stable operation for 100 h at 400 mA cm^−2^, 55 °C, and ambient pressure, delivering a cell voltage of 1.98 V and an energy consumption as low as 4.7 kWh N m^−3^ H_2_ [151]. By effectively addressing the challenges faced by both PEM and conventional alkaline electrolyzers in direct seawater electrolysis, the AEM electrolyzers offer an economical, flexible, and scalable solution. These advantages position AEM electrolyzers as a highly promising technology for clean energy conversion and broader utilization.

As a promising candidate for direct seawater electrolysis, the AEM electrolyzer presents significant application potential. However, it encounters numerous technical challenges in achieving commercial viability. A critical issue is the selective permeability of the AEM, which is essential for screening ions and preventing cations from permeating the cathode. Insufficient selectivity may result in the deposition or passivation of metal hydroxides, thereby impairing the functionality of active sites (Fig. 8b) [156]. Despite the high costs associated with PEM water electrolysis devices, they serve as an excellent example of superior ion-selective permeability [157]. The PEM electrolyzer maintains an optimal pH environment for HER due to the high concentration of H⁺ ions facilitated by the membrane. As depicted in Fig. 8c, this membrane serves as an effective filtration barrier, isolating the cathode from seawater impurities and protecting it from detrimental interference, which enhances the efficiency and stability of the electrolysis process [77].

During seawater electrolysis, the ClER occurs at the anode of AEM electrolyzers, leading to severe electrode corrosion, while the cathode, despite lacking competitive reactions with HER, still faces challenges from Cl_2_ corrosion [79]. The long-term stability of the system during seawater electrolysis can be significantly enhanced by incorporating a selective barrier layer on the catalyst, such as MnO_x_[101], Cr(OH)_3_[158], or graphite shells [159]. The presence of a selective barrier layer can restrict the involvement of undesired Cl^−^ in chemical reactions at the anode. Similarly, this principle can be applied to protect the cathode from corrosion caused by Cl^−^ during seawater electrolysis, as well as preventing the agglomeration of impurity ions and the poisoning of the catalyst (Fig. 8d). However, this strategy may be limited by mass transfer efficiency and requires further optimization to ensure a high-performance electrolysis process. Moreover, although AEM electrolyzers are designed to minimize start-stop losses, frequent cycling and power adjustments can still induce wear on the system. Despite their relatively low maintenance costs, additional optimization is necessary to mitigate potential losses and improve the operational efficiency and longevity of the electrolyzer.

### Novel Seawater Electrolysis Systems

**Fig. 9:**
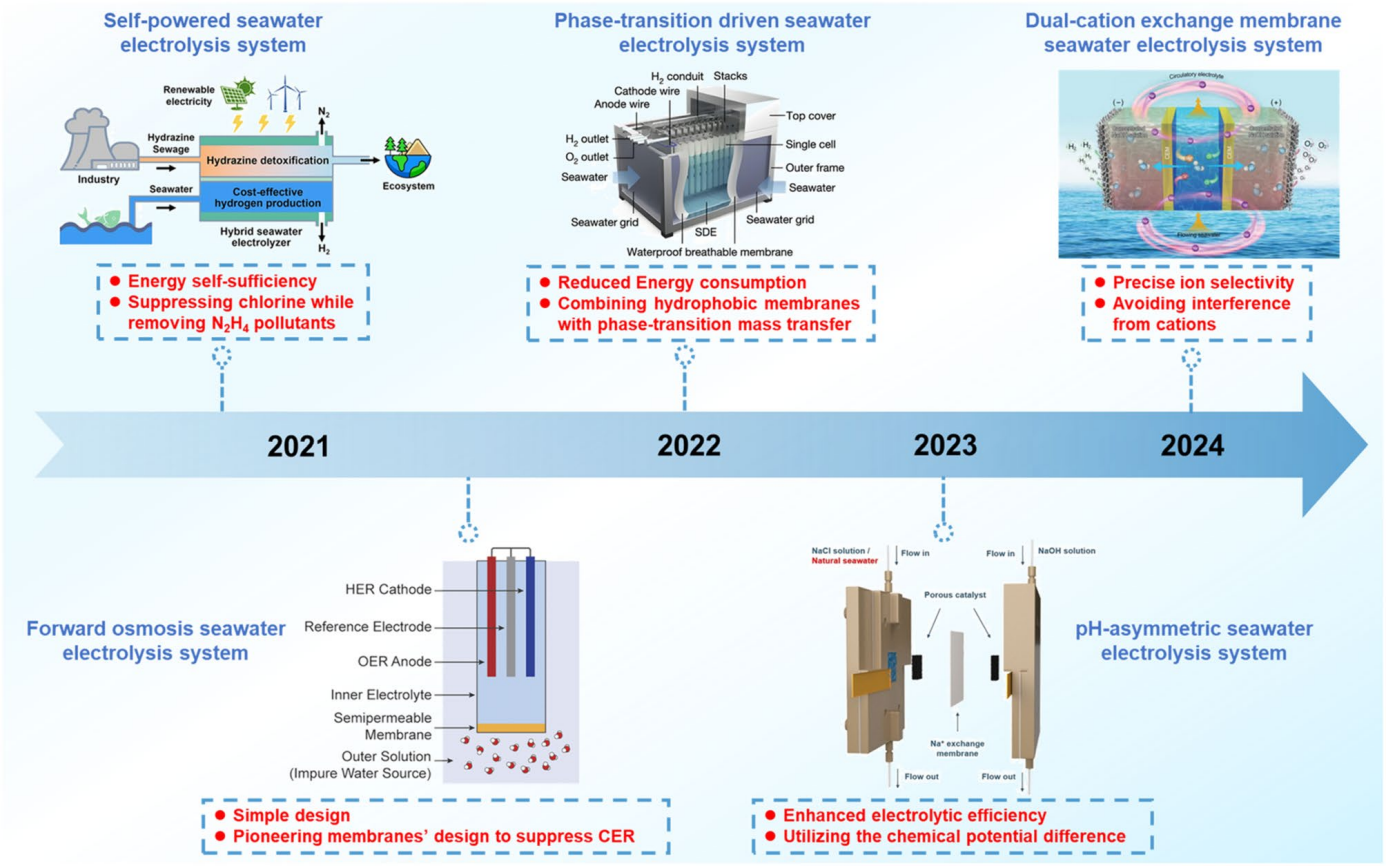
Recent advances in seawater electrolysis systems over the past five years

Although AEM electrolyzers demonstrate significant advantages in seawater electrolysis, such as high adaptability and the use of non-precious metal catalysts, they still confront persistent challenges including Cl^−^ permeation and cationic corrosion, which hinder their broader application in cost-effective and sustainable hydrogen production [31, 70]. To overcome these limitations, researchers are developing innovative chlorine-free, energy-efficient seawater electrolysis technologies aimed at mitigating Cl^−^ interference and cationic corrosion while minimizing energy consumption, thereby enhancing overall electrolysis efficiency and economic feasibility. This section reviewed several emerging seawater electrolysis systems over the past five years, including self-powered seawater electrolysis systems, forward osmosis seawater electrolysis systems, phase-transition-driven seawater electrolysis systems, pH-asymmetric seawater electrolysis systems, and dual-cation exchange membrane seawater electrolysis systems, as shown in Fig. 9.

Understanding these advancements sheds light on the progress made in seawater electrolysis technology. It also emphasizes their role in achieving economically viable and sustainable hydrogen production, which is crucial for advancing the hydrogen economy.

#### Self-Powered Seawater Electrolysis System

Qiu et al. pioneered a self-powered hybrid seawater electrolysis technology by integrating solar cells, introducing an innovative approach to convert marine resources into clean hydrogen fuel and simultaneously removing N_2_H_4_ pollutants from the wastewater [160]. Coupling seawater electrolysis with the hydrazine oxidation reaction (HzOR) leverages the lower thermodynamic potential of HzOR, enabling hydrogen production at a reduced cell voltage. This method offers two primary advantages. Firstly, since the potential of HzOR is significantly lower than that of chlorine oxidation, it effectively avoids chlorine chemistry issues. This minimizes the generation of hazardous chlorine compounds. Importantly, it does not compromise electrolysis current or hydrogen production efficiency [161, 162]. Secondly, this approach bypasses energy-intensive OERs, reducing external power requirements and facilitating integration with solar cells. Additionally, electrocatalytic HzOR efficiently removes hydrazine from industrial wastewater without the need for extra oxidants or complex separation processes, enhancing the environmental sustainability of the system and economic viability [163].

**Fig. 10:**
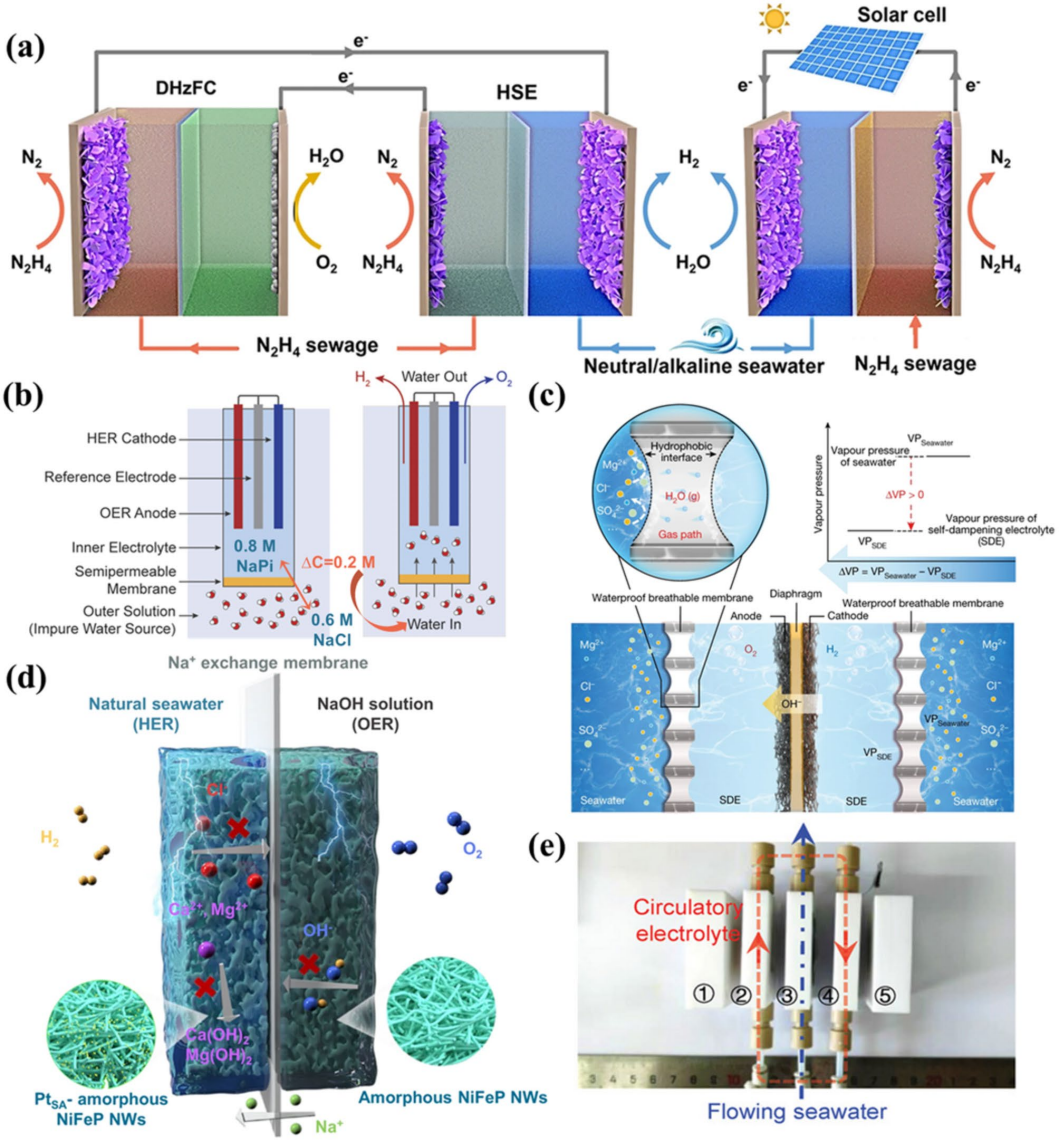
**a** Schematic diagram of a self-powered hydrogen production system by integrating a hybrid seawater electrolyzer into a low-pressure direct hydrazine fuel cell (DHzFC) or a solar cell [160]. **b** Schematic diagram of FOSE electrolysis system composition and structural principle [165]. **c** Schematic diagram of the migration mechanism and migration process of water purification based on the liquid–vapor-liquid phase transition [27]. **d** Design scheme of a pH-asymmetric electrolyzer for the Na^+^ exchange membrane [167]. **e** Schematic of CEM three-compartment electrolyzer structure [69]

The core of self-powered electrolysis lies in coupling the electrolysis process with solar cells to achieve energy autonomy **(**Fig. 10a). By incorporating a low-voltage hydrazine fuel cell or solar cell, the system utilizes solar power during periods of adequate sunlight, reducing reliance on external power grids. The solar cell not only powers the electrolysis but also stores energy through water splitting or hydrazine degradation, enabling continuous hydrogen production during low solar conditions. This integration significantly improves the system energy efficiency, with reduced energy consumption by 40% ~ 50% compared to conventional alkaline water electrolysis and reduced carbon emissions by over 90% compared to hydrogen production via natural gas reforming [16, 164]. However, the efficiency and sustainability of self-powered systems are critically dependent on the performance and durability of the solar cells. Furthermore, the complex design and maintenance of self-powered systems require precise coordination among all components and their adaptability to varied environmental conditions to maintain consistent operational stability.

#### Forward Osmosis Seawater Electrolysis System

In comparison with self-powered seawater electrolysis systems, the forward osmosis seawater electrolysis (FOSE) system offers a streamlined alternative to self-powered seawater electrolysis, employing a semipermeable cellulose acetate membrane to mitigate chlorination reactions in the seawater. This design harnesses a concentration gradient to drive water molecules from a saline source (brine or seawater) into a more concentrated electrolyte, with the gradient maintained by the dissociation of water molecules, balancing the rates of inflow (forward osmosis) and outflow (water splitting) [165].

As illustrated in Fig. 10b, the system integrated an acetate cellulose semipermeable membrane within the electrolyzer to partition the 0.8 M NaPi (Pi, phosphate) internal electrolyte from the external 0.6 M NaCl solution, establishing a 0.2 M concentration gradient conducive to the electrochemical water-splitting process. This configuration sustained a nearly constant salt concentration in the external solution, providing stable osmotic pressure. During electrolysis, the decomposition of water into H_2_ and O_2_ drove the outward flow of water, perpetuating the concentration gradient that continuously drew water molecules from the saline source via forward osmosis, supplying purified water for subsequent splitting [165].

The FOSE system facilitates a balanced and continuous inflow and outflow of water molecules, enabling HER and OER to achieve unit FE while preventing Cl^−^ permeation, thereby averting ClER-induced corrosion in direct seawater electrolysis. Operating with conventional, stable electrodes, the system efficiently separates water from saline sources at high current densities, eliminating the need for additional purification and desalination processes. The FOSE system thus provides a simplified pathway for selective hydrogen and oxygen generation from saline water, minimizing energy efficiency losses. However, while the composition of the FOSE system is relatively straightforward, its long-term stability and durability in practical applications require further validation through extensive testing.

#### Phase-Transition-Driven Seawater Electrolysis System

In the FOSE system, although the hydrophobic semipermeable membrane was engineered to block impurity ions from migrating from seawater to the electrolyte, significant ion cross-diffusion still occurs, indicating poor ion selectivity [122, 166]. This necessitates the use of a neutral electrolyte to maintain membrane stability, which sacrifices the performance of the electrolysis. In contrast, the phase-transition-driven seawater electrolysis system developed by Xie et al. employed a hydrophobic polytetrafluoroethylene (PTFE) membrane as the gas pathway interface, coupled with a concentrated KOH solution as a self-wetting electrolyte (SDE). This setup leveraged a liquid–gas–liquid phase transition for mass transfer, significantly enhancing the efficiency of the electrolysis. The perfluorinated structure of the PTFE membrane imparted low surface energy, establishing a superhydrophobic barrier that effectively suppressed seawater and ion permeation, leading to more stable and efficient long-term electrolysis performance [27].

During operation, the pressure difference of the water vapor between the seawater and the SDE-driven electrolysis cell propels the spontaneous gasification of the seawater. Water vapor diffused through the specially designed short gas passage in the membrane to the SDE side, where it was absorbed and re-liquefied, producing pure water directly at the seawater source and achieving complete ion blocking. This continuous water consumption by electrolysis sustained the vapor pressure differential across interfaces. When the migration rate of water matched with the electrolysis rate, a new thermodynamic equilibrium was established between the seawater and the SDE, perpetuating a stable “liquid-air-liquid” phase change process that supplied freshwater for electrolysis, as depicted in Fig. 10c.

The integration of the PTFE hydrophobic membrane effectively prevents the seawater and impurity ion permeation, enhances self-sufficiency by minimizing dependency on external water sources via the SDE, and substantially reduces energy losses through a precisely designed liquid-gas-liquid phase transition mass transfer mechanism. It includes strategic membrane material selection, optimized electrolyte configuration, and advanced mass transfer process regulation. These factors work together to enhance the efficiency of hydrogen generation. They also improve the overall stability of the system. PTFE membranes initially exhibit high performance due to their excellent hydrophobicity and resistance to contamination. However, there is a potential risk of membrane wetting and fouling during prolonged operation. This risk necessitates thorough evaluation to ensure that the complex chemistry of seawater does not gradually degrade membrane performance.

#### pH-Asymmetric Seawater Electrolysis System

Li et al. developed a pH-asymmetric electrolysis system, which is similar to phase-transition-driven seawater electrolysis using selective permeable membrane technology, effectively reduces the voltage of the electrolysis, and enhances efficiency by exploiting the chemical potential differences between electrolytes with varying pH values. Unlike the design of the hydrophobic PTFE membrane in phase-transition-driven systems, this system operates by separating the electrolyzer chambers using a Na^+^ exchange membrane, which selectively allows Na⁺ ions to pass while blocking Cl^−^ and other ions [167]. This maintains pH asymmetry between the chambers and also avoids the issue of PTFE membrane wetting during long-term operation. As depicted in Fig. 10d [168], the cathode chamber is circulated with NaCl solution or natural seawater, while the anode chamber uses NaOH solution. At the cathode, water dissociates more efficiently into H^+^ and OH^−^ at lower pH, whereas the high pH facilitates the combination of dissociated H^+^ and OH^−^ to form water at the anode, thus promoting continuous water splitting.

The advantages of this pH-asymmetric electrolysis lie in its significant reduction of energy consumption and enhancement of efficiency by utilizing chemical potential gradients, allowing operation at lower voltages and minimizing energy input. The Na^+^ exchange membrane effectively mitigates Cl^−^-induced corrosion and side reactions. Furthermore, it reduces the precipitation of ions like Ca^2+^ and Mg^2+^ at the anode, preventing the blockage of active sites. This design preserves the active surface of the electrode, leading to an increase in the FE. It also significantly reduces energy consumption and maintenance costs during seawater electrolysis for hydrogen production. Although the pH-asymmetric electrolysis system features a relatively simple design, practical implementation requires more sophisticated system integration and optimization to adapt to diverse seawater conditions and operational environments.

#### Dual-Cation Exchange Membrane Seawater Electrolysis System

Although Na^+^ exchange membranes effectively inhibit Cl^−^ migration from seawater to the electrolyte, the precipitation of Mg^2+^ and Ca^2+^ still constrains their practical application in pH-asymmetric seawater electrolyzers [169]. To overcome this limitation, Cui et al. developed a dual-cation exchange membrane (DCEM) three-compartment electrolyzer, incorporating a recirculating electrolyte system to facilitate continuous hydrogen production. This configuration employs monovalent selective DCEMs to maintain ionic neutrality during electrolysis, effectively mitigating interference from Mg^2+^, Ca^2+^, and Cl^−^ ions [69].

As illustrated in Fig. 10e, the electrolyzer is structured with an anode, cathode, and intermediate chamber, and each component is separated by specialized dual-cation exchange membranes. In Fig. 10e, the areas designated as 2 and 4 both correspond to nickel foam electrodes, and the area marked as 3 is a bipolar membrane. The nickel foam electrodes and the bipolar membrane together form the anode and cathode chambers of the electrolytic cell. The anode chamber circulates seawater or an electrolyte-containing solution, while the cathode chamber employs an alkaline solution. Under electric current, water dissociates in the cathode chamber, generating H_2_ and OH^−^; the H_2_ is collected, and OH^−^ migrates through the exchange membrane to the intermediate or anode chamber. In the anode chamber, water molecules undergo oxidation, forming O_2_ and H^+^, which then combine with OH^−^ to reform water, maintaining the ionic equilibrium. The self-circulating mechanism of the system stabilizes electrolyte concentration and pH, ensuring continuous and stable electrolysis.

Compared to conventional single-membrane systems, the DCEM electrolyzer enhances the efficiency and stability of the electrolysis by optimizing ion selectivity and transport process, improving water and ion management, minimizing water loss and side reactions, and thus boosting energy conversion efficiency. However, the complexity of the three-compartment system may necessitate regular maintenance and monitoring to ensure long-term stability, potentially increasing operational complexity and maintenance costs.

To effectively utilize abundant seawater resources, electrolytic systems have been the focus of extensive research in recent years. Among these, AEM electrolyzers, characterized by their alkaline operating conditions and compatibility with non-precious metal catalysts, exhibit a strong synergy with renewable energy sources. This synergy enables precise regulation of fluctuating energy inputs, enhancing energy efficiency, and expanding their applicability in seawater electrolysis. However, they still face challenges, particularly the need for improved corrosion resistance to Cl^−^ and reduced energy consumption. Recent advancements in seawater electrolysis systems incorporate innovative membrane technologies and reactor designs, effectively mitigating Cl^−^-induced corrosion and side reactions. Self-powered seawater electrolysis systems achieve energy autonomy while effectively eliminating N_2_H_4_ pollutants and suppressing chlorine production; however, their complex system design presents significant challenges. To address this issue, researchers have developed the forward osmosis seawater electrolysis system that simplifies design but is constrained by limitations in membrane ion selectivity. Additionally, the phase-transition-driven seawater electrolysis system utilizes a PTFE hydrophobic membrane to inhibit ion permeation effectively, though concerns arise regarding membrane wetting and potential performance degradation during prolonged operation. To enhance efficiency, the Na^+^ cation exchange membrane system has been engineered to leverage chemical potential differences, yet its anti-fouling capabilities remain limited. To further mitigate fouling from cation precipitation in seawater, the dual-cation exchange membrane seawater electrolysis system has been introduced, which not only suppresses chlorine production but also demonstrates improved resistance to fouling.

## Conclusion and Outlook

Recent advance aimed at improving the efficiency and stability of seawater electrolysis, including the development of novel catalytic materials, the use of electrolyte additives, and the optimized membrane designs. Among various electrolyzer technologies, AEM electrolyzers stand out due to their utilization of cost-effective catalytic materials and compatibility with renewable energy sources. However, AEM systems are still susceptible to Cl^−^ corrosion, necessitating the development of advanced seawater electrolysis systems, such as self-powered, forward osmosis, and phase-transition-driven systems. These innovative designs not only suppress Cl^−^ corrosion and side reactions but also offer new strategies for enhancing electrolysis efficiency and reducing energy consumption.

Significant progress has been made in the development of efficient seawater electrolysis technologies. However, to ensure the viability of this approach, a comprehensive assessment of the economic and environmental impacts of large-scale seawater electrolysis is essential, including potential ecological damage and the energy required for seawater desalination. Recent research on direct seawater electrolysis has often overshadowed these practical considerations, leading to misallocation of resources and potentially delaying more feasible and direct green hydrogen solutions. Crucially, a thorough evaluation of the technical and economic feasibility of direct seawater electrolysis compared to traditional seawater desalination-based hydrogen production is required, particularly in terms of the energy savings and cost implications associated with seawater desalination. The focus should be on improving the overall efficiency and durability of the electrolysis system, rather than solely on the direct use of seawater. As the field continues to advance, achieving sustainable and cost-effective large-scale hydrogen production will necessitate a balanced approach that integrates both scientific innovation and practical implementation. To accomplish this, several challenges need to be overcome to ensure the viability of this approach:

1. Construction of Chloride-Utilizing Catalysts: Cl^−^ ions are widely recognized as the predominant contributors to the degradation of electrocatalyst active sites. Nevertheless, strategically leveraging Cl^−^ ions from seawater to enhance OER activity could pave a transformative pathway for future catalyst design. Qiao et al. exploited the auxiliary role of Cl^−^ ions to drive the transfer of active sites from Ru to Mn, forming a negatively charged hydroxyl (^*^OH) layer on the Mn surface, which shielded the catalyst from Cl^−^-induced corrosion [83]. This strategy transforms Cl^−^ ions from a harmful agent into a promoter of OER performance. The development of multi-component catalysts with multiple active sites, each capable of interacting with Cl^−^ ions at distinct locations to facilitate active site conversion, presents extensive application potential.
2. Catalysts engineering aligning with novel systems: Designing high-performance catalytic materials tailored for innovative systems could achieve multiple critical objectives. For instance, a recent design by Xie et al. of an Fe–Ni(OH)_2_/NF catalyst facilitated the chemical reduction of [Fe(CN)_6_]^3−^, leading to oxygen evolution [106]. This approach spatially and temporally decouples the HER and OER, with mitigating Cl^−^-induced corrosion, and they also addressed the high-voltage demands and gas purity challenges. The use of high-efficiency catalytic materials accelerates electrolysis kinetics, and when integrated with advanced system architectures, it further reduces the required electrolysis voltage due to the enhanced conductivity and optimized mass transfer of reactants and products. This synergy enhances overall efficiency and reduces energy consumption, paving the way for more economical and environmentally sustainable seawater electrolysis technologies.
3. Application of novel electrolyte additives: Although current additives, including sulfate and phosphate compounds, partially mitigate chloride-induced corrosion, cation-induced corrosion remains a challenge. Thus, designing new electrolyte additives capable of inhibiting cation-induced damage is vital for enhancing system stability and performance. As new electrolyte additives, hard Lewis acid materials are capable of dissociate water molecules and effectively sequester OH^−^, thereby generating a localized alkaline microenvironment around catalyst [82]. This microenvironment significantly enhances the HER kinetics at the cathode and simultaneously prevents the formation of insoluble precipitates that result from the interaction between cations (e.g., Mg^2+^ and Ca^2+^) and OH^−^. The deployment of such advanced additives will be a pivotal direction for the future of seawater electrolysis.
4. Advancements in ion-selective and stabilizing membranes: The manufacturing of membranes with high ion selectivity and exceptional chemical stability is imperative for the sustained and efficient operation of direct seawater electrolysis systems. The high ion selectivity in membranes requires the presence of ion-conducting channels with precise dimensions and chemical properties that selectively permit the passage of OH^−^ against Cl^−^. These channels are typically covered with functional groups, including sulfonic acid, carboxylic acid, or quaternary ammonium groups, embedded within the membrane matrix [170]. The highly cross-linked polymer network could enhance the chemical stability of the membrane [171], ensuring durability in alkaline conditions and resistance to corrosive substances in seawater. If the membrane structure cannot simultaneously achieve high ion selectivity and stability, the incorporating reinforcements such as carbon fibers, glass fibers, alumina, or silica nanoparticles, etc., could enhance stability [172]. These reinforcements, uniformly dispersed within the polymer matrix, bolster the overall performance of the membrane.

These strategies collectively highlight the pathway toward overcoming existing challenges and enhancing the performance, stability, and commercial viability of direct seawater electrolysis systems.
